# Supplementary material for: Mouse Slfn8 and Slfn9 genes complement human cells lacking SLFN11 during the replication stress response
Source: Commun Biol. 2023 Oct 13;6:1038. doi: 10.1038/s42003-023-05406-9 (PMC10575959; doi:10.1038/s42003-023-05406-9)
Supplement: Supplementary file 2 — Supplementary Information [file 42003_2023_5406_MOESM2_ESM.pdf]

Supplementary Information

Mouse *Slfn8* and *Slfn9* genes complement human cells lacking *SLFN11* during the replication stress response

Erin Alvi[1,$], Ayako L. Mochizuki[1,+], Yoko Katsuki[1,#], Minori Ogawa[1], Fei Qi[1], Yusuke Okamoto[1,*], Minoru Takata[1,2], Anfeng Mu[1,2,**]

[1]Laboratory of DNA Damage Signaling, Department of Late Effects Studies, Radiation Biology Center, Graduate School of Biostudies, Kyoto University, Kyoto, Japan
[2]Multilayer Network Research Unit, Research Coordination Alliance, Kyoto University, Kyoto, Japan
**To whom correspondence should be addressed: mu.anfeng.7x@kyoto-u.ac.jp
[$]Current Address: Laboratory of Biochemical Cell Dynamics, Institute for Integrated Cell-Material Sciences (WPI-iCeMS), Graduate School of Biostudies, Kyoto University, Kyoto, Japan.
[+]Current address: CiRA Foundation, Kyoto, Japan
[#]Current address: Department of Cellular Biochemistry, Graduate School of Pharmaceutical Sciences, Kyushu University, Fukuoka, Japan.
*Current address. Lunenfeld-Tanenbaum Research Institute, Mount Sinai Hospital, Toronto, Ontario, Canada.

This PDF file incudes:

Supplementary Figures 1 to 13
Supplementary Tables 1, 2

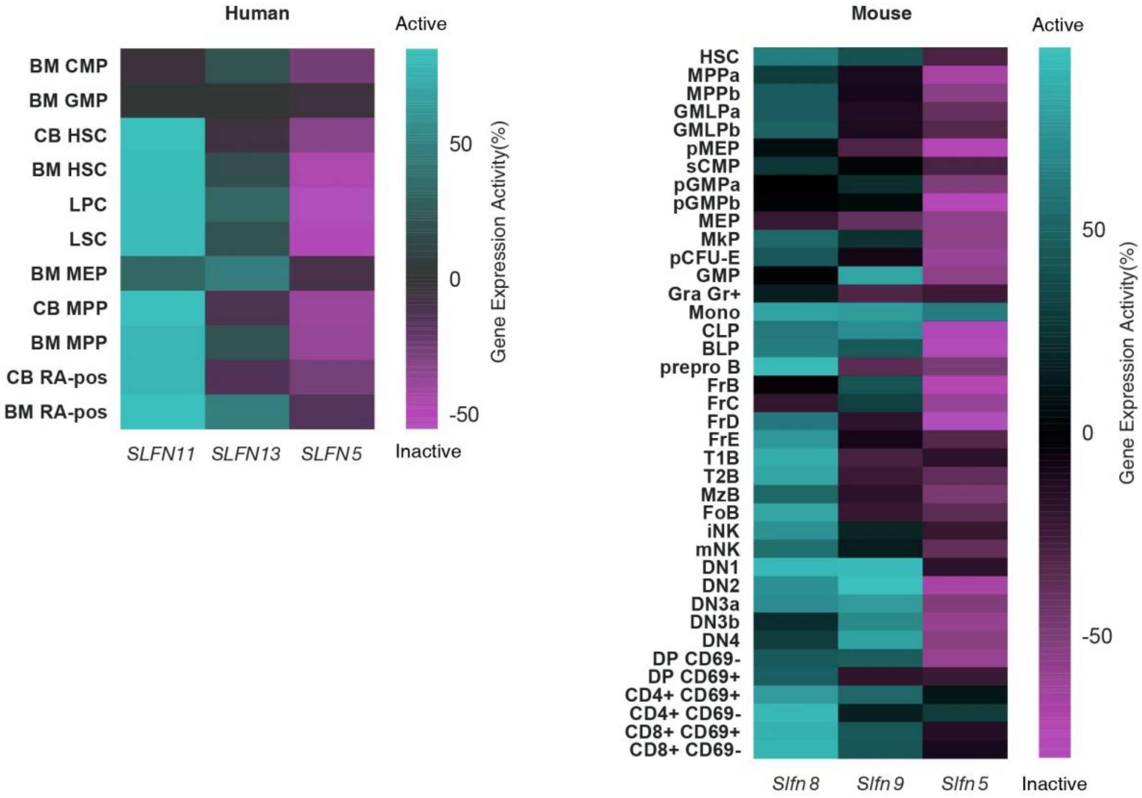

Supplementary Figure 1. **Expression of subgroup III *SLFNs* of human and mouse during hematopoiesis**. Relative expression levels of *SLFNs* in sorted subpopulations of human or mouse hematopoietic tissue, as determined by the Affymetrix microarray analysis. The data are derived from, and accessible through, the Gene Expression Commons platform (https://gexc.riken.jp)[1]. In Gene Expression Commons, raw microarray data from various experiments are individually mapped against a “Common Reference”, a large-scale (> 10,000) pooled data set, enabling absolute gene expression profiling. The high or low expression levels (avtivity) are expressed as a percentile in a high or low range, and indicated by the color gradiation as the highest (+100%) in cyan green, and the lowest (-100%) as dark violet. For futher details, see Seita J. et al. *PloS one* 2012 [1]. BM, bone marrow; CB, cord blood; CMP, common myeloid progenitor; GMP, granulocyte/monocyte progenitor; MEP, megakaryocyte/erythroid progenitor; HSC, hematopoietic stem cell; LPC, non-stem leukemia progenitor cell; LSC, leukemia stem cell; MEP, megakaryocyte-erythroid progenitors; MPP, multipotent progenitor cells; RA-pos, CD45RA positive cell; GMLP, granulocyte-monocyte-lymphoid progenitor; MkP, megakaryocyte-committed progenitor; CFU-E, colony-forming unit-erythroid; BLP, B lymphoid progenitors; CLP, common lymphoid progenitor. For additional nomenclature of mouse hematopoietic subpopulations, see Seita et al.[1]

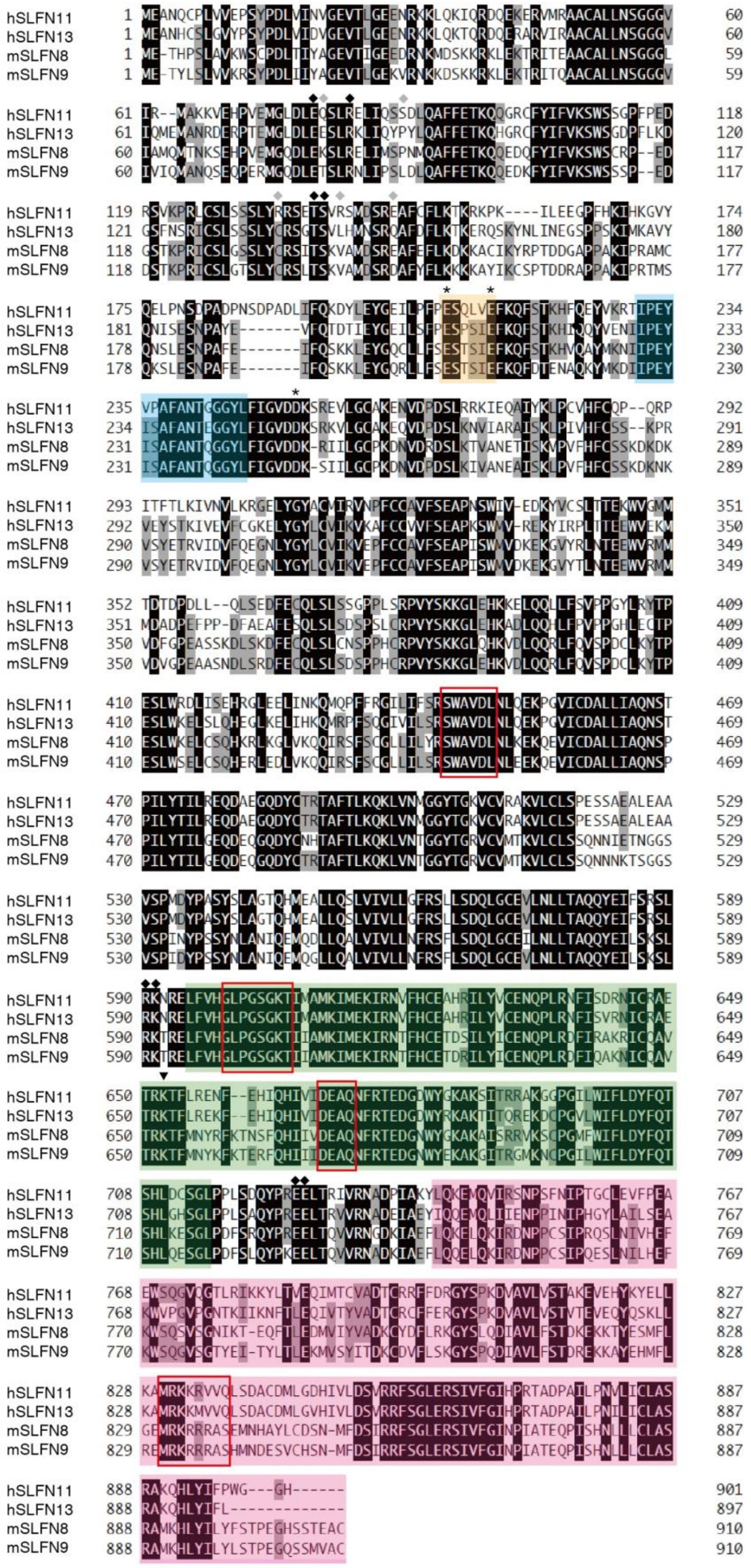
hSLFN11
hSLFN13
mSLFN8
mSLFN9

Supplementary Figure 2. MAFFT alignments of human SLFN11 and mouse SLFN8 and 9. Each amino acid residue is grey shaded if three out of four are the same or in the black box if all four are the same. The blue area indicates the SLFN box. The yellow box indicates the putative catalytic region of the RNase domain. ⁎ : the two glutamic acid and one aspartic acid residues critical for the RNase enzymatic activity[2,3]. ▼ : ssDNA binding site[4]. ◆ : conserved residues between SLFN11 and SLFN8 or SLFN9 in the SLFN11 dimer interface including interface I (R590 to E726, K591 to E725) and interface II (E78, R82, T138, S139, and E147). ◆: Q79, S88, R134, and R141 are not conserved [4]. The green area represents the putative helicase domain. The red box indicates conserved residues in the SWAVDL domain and the Walker type A and B motifs in the helicase domain. The purple box represents the predicted NLS in mSLFN8/9 and hSLFN11. The pink area represents the RPA interacting region[5]. The method used for alignment was the MAFFT program (Genetyx -Mac). The conserved regions were from the Pfam database and the literature[6,7].

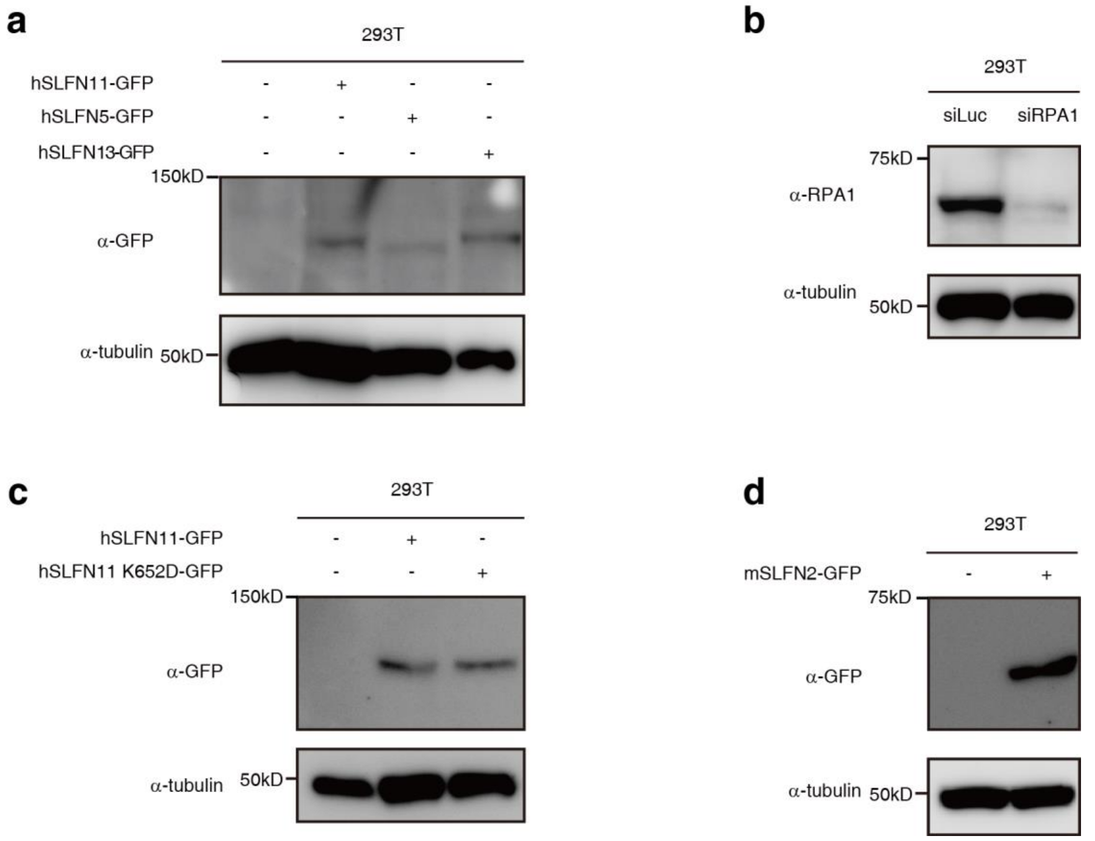

Supplementary Figure 3. Western blotting analysis of SLFNs. a GFP-tagged human SLFN11, 5, and 13 expression plasmids were verified by transfecting into 293T cells and western blotting. **b** siRPA1 was verified by transfecting into 293T cells and western blotting with siLuciferase (siLuc) as a control. **c** GFP-tagged human SLFN11 K652D or **d** mouse SLFN2 expression plasmids were verified by transfecting into 293T cells and western blotting.

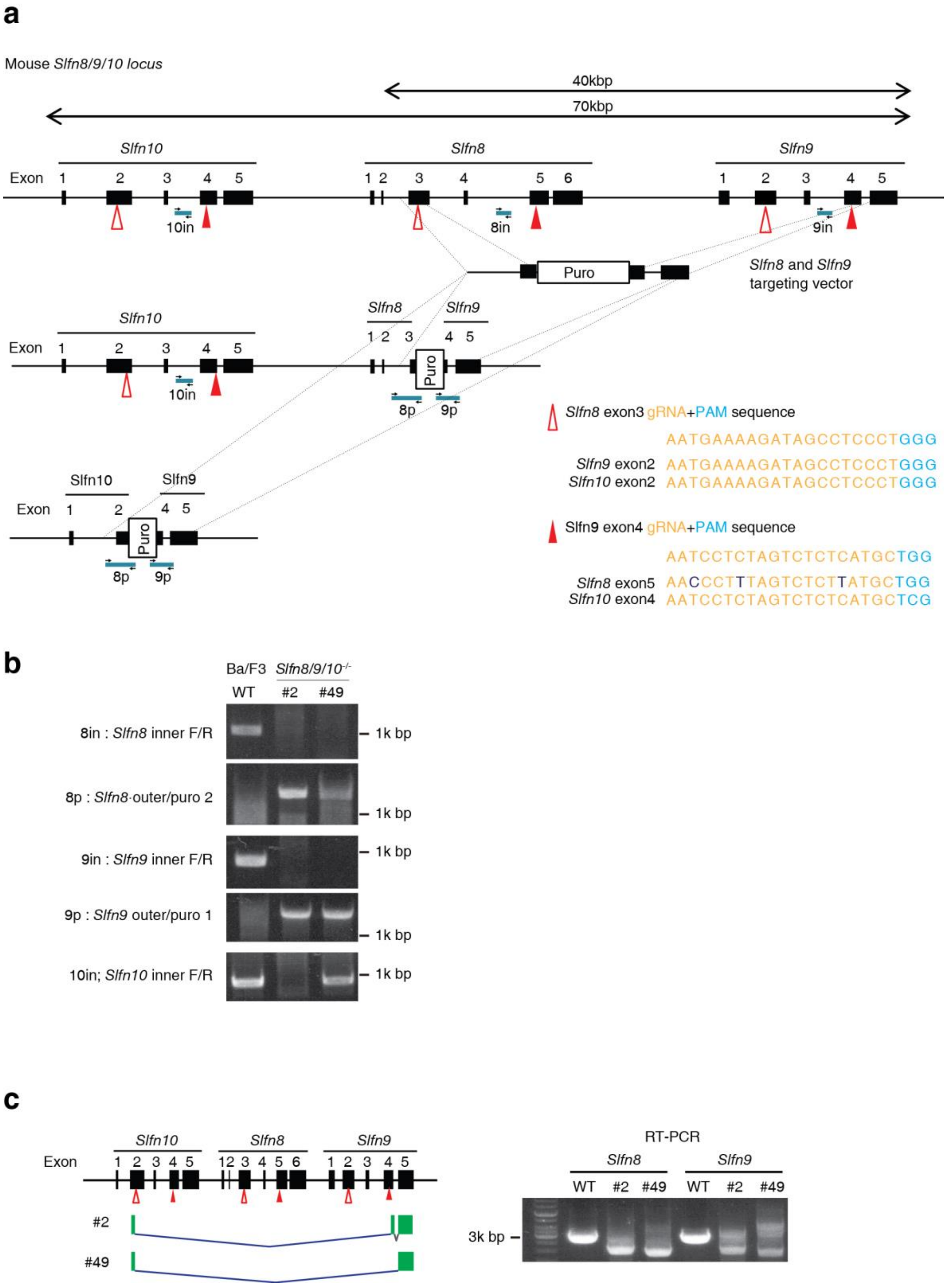
a
Mouse Slfn8/9/10 locus
40kbp
70kbp
Slfn10
Slfn8
Slfn9
Exon
1 2 3 4 5
1 2 3 4 5 6
1 2 3 4 5
10in
8in
9in
Puro
Slfn8 and Slfn9 targeting vector
Slfn10
Slfn8
Slfn9
Exon
1 2 3 4 5
1 2 3
4 5
Puro
10in
8p
9p
Slfn8 exon3 gRNA+PAM sequence
AATGAAAAGATAGCCTCCCTGGG
Slfn9 exon2 AATGAAAAGATAGCCTCCCTGGG
Slfn10 exon2 AATGAAAAGATAGCCTCCCTGGG
Slfn10
Slfn9
Exon 1 2 4 5
Puro
8p
9p
Slfn9 exon4 gRNA+PAM sequence
AATCCTCTAGTCTCTCATGCTGG
Slfn8 exon5 AACCCTTTAGTCTCTTATGCTGG
Slfn10 exon4 AATCCTCTAGTCTCTCATGCTCG
b
Ba/F3
Slfn8/9/10-/-
WT #2 #49
8in : Slfn8 inner F/R
1k bp
8p : Slfn8 outer/puro 2
1k bp
9in : Slfn9 inner F/R
1k bp
9p : Slfn9 outer/puro 1
1k bp
10in; Slfn10 inner F/R
1k bp
c
Slfn10
Slfn8
Slfn9
Exon
1 2 3 4 5
12 3 4 5 6
1 2 3 4 5
#2
#49
RT-PCR
Slfn8
Slfn9
WT #2 #49
WT #2 #49
3k bp

Supplementary Figure 4. Generation of Ba/F3 *Slfn8/9* knockout cell line. **a** A schematic showing the gene targeting strategy for *Slfn8* and *Slfn9*. The wild type regions spanning ~70 kb genome encompassing *Slfn8*, *Slfn9*, and *Slfn10* genes (upper), the expected configuration in which *Slfn8* and *9* were targeted (middle, *Slfn10* was intact in this case) or *Slfn8, 9, 10* were simultaneously disrupted (lower) were shown. The *Slfn8* and *Slfn9* targeting vector contained part of the *Slfn8* and *Slfn9* genes as upper and lower arms and the puromycin resistance gene cassette as shown. Because of the highly homologous sequences in *Slfn8/9/10* genes, the designed CRISPR-CAS9 cleavage sites in *Slfn8* exon3 (indicated by open red triangles) or *Slfn9* exon 4 (closed red triangles) also existed in the other genes as indicated (three mismatches exist in *Slfn8* gene for *Slfn9* CRISPR). Ba/F3 cells were simultaneously transfected with the targeting vector and two CRISPR plasmids, and selected by puromycin. **b** Genomic PCR of the wild type and knockout clones #2 and #49. Positions of each amplicon are indicated by green lines in **a**. PCR primer pairs are indicated on the left side of the gel image. 8in, 9in, or 10in are PCRs detecting *Slfn8*, *Slfn9* or *Slfn10* deletions by integration of the targeting vector, respectively. 8p and 9p PCRs are designed to detect the integration of the targeting vector with an outside primer and a *puro* primer. **c** Reverse transcription (RT)-PCR analysis of the knockout clones. In clone #2, *Slfn10* gene was also targeted, while clone #49 retained at least some part of *Slfn10* intact. However, Sanger sequencing of RT-PCR products confirmed that *Slfn10* gene in the clone #49 was destroyed. Primer sequences are provided in the Supplementary Table S2.

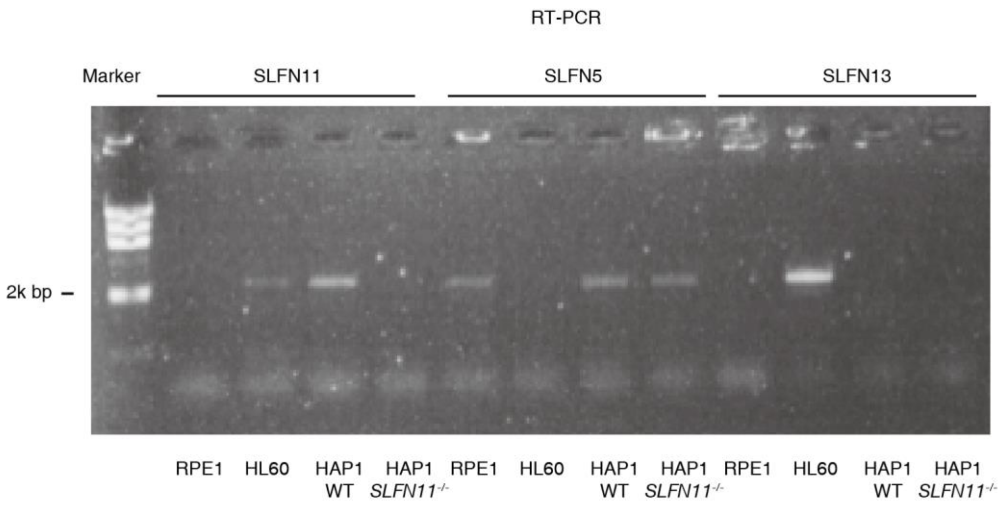

Supplementary Figure 5. RT-PCR analysis of human *SLFNs* with indicated cell lines. Total RNA was isolated from indicated cell lines and cDNA was synthesized. The full-length coding sequences were amplified with KOD-one polymerase. The molecular marker was λHindIII.

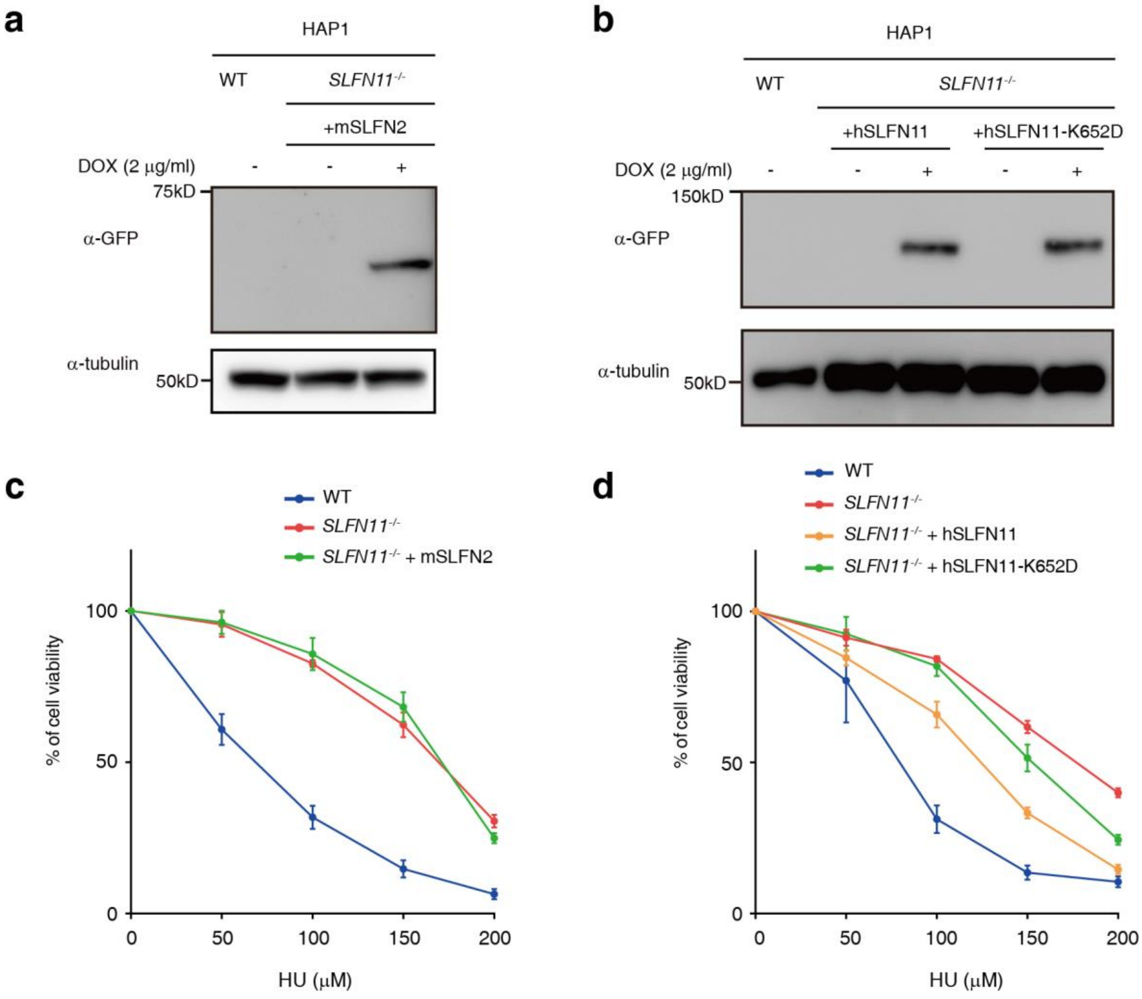

Supplementary Figure 6. Analysis of *SLFNs* function in human HAP1 cell lines. Western blotting analysis of HAP1 *SLFN11*$^{-/-}$ cell line with DOX-induced expression of **a** mSLFN2-GFP, **b** hSLFN11-K652D. HU sensitivity of the HAP1 cell lines with **c** mSLFN2 or **d** hSLFN11 K652D expression. Mean ± SD in quadruplicate cultures is shown.

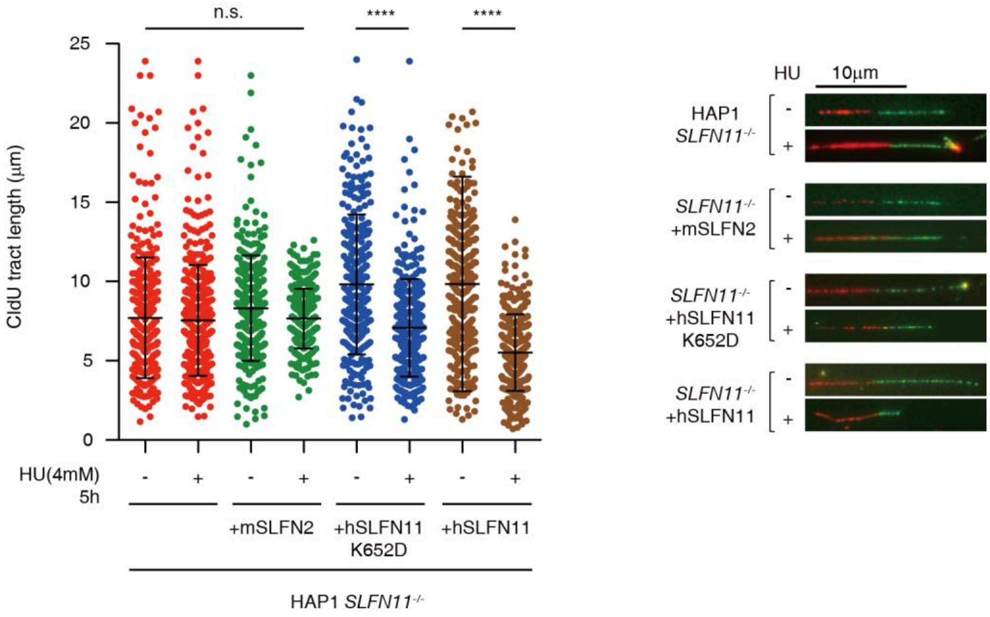

Supplementary Figure 7. DNA fiber assay analysis of SLFNs expression in HAP1 *SLFN11*$^{-/-}$ cells. For each sample, the length of 300 CldU tracts was measured. The P values were calculated using one-way ANOVA with Tukey's multiple-comparisons test. To minimize observer bias, the images were captured and analyzed in a blinded manner. Represent images are shown. Mean ± SD (n≥300) are shown. n.s. : not significant.
**** : $p<0.0001$.

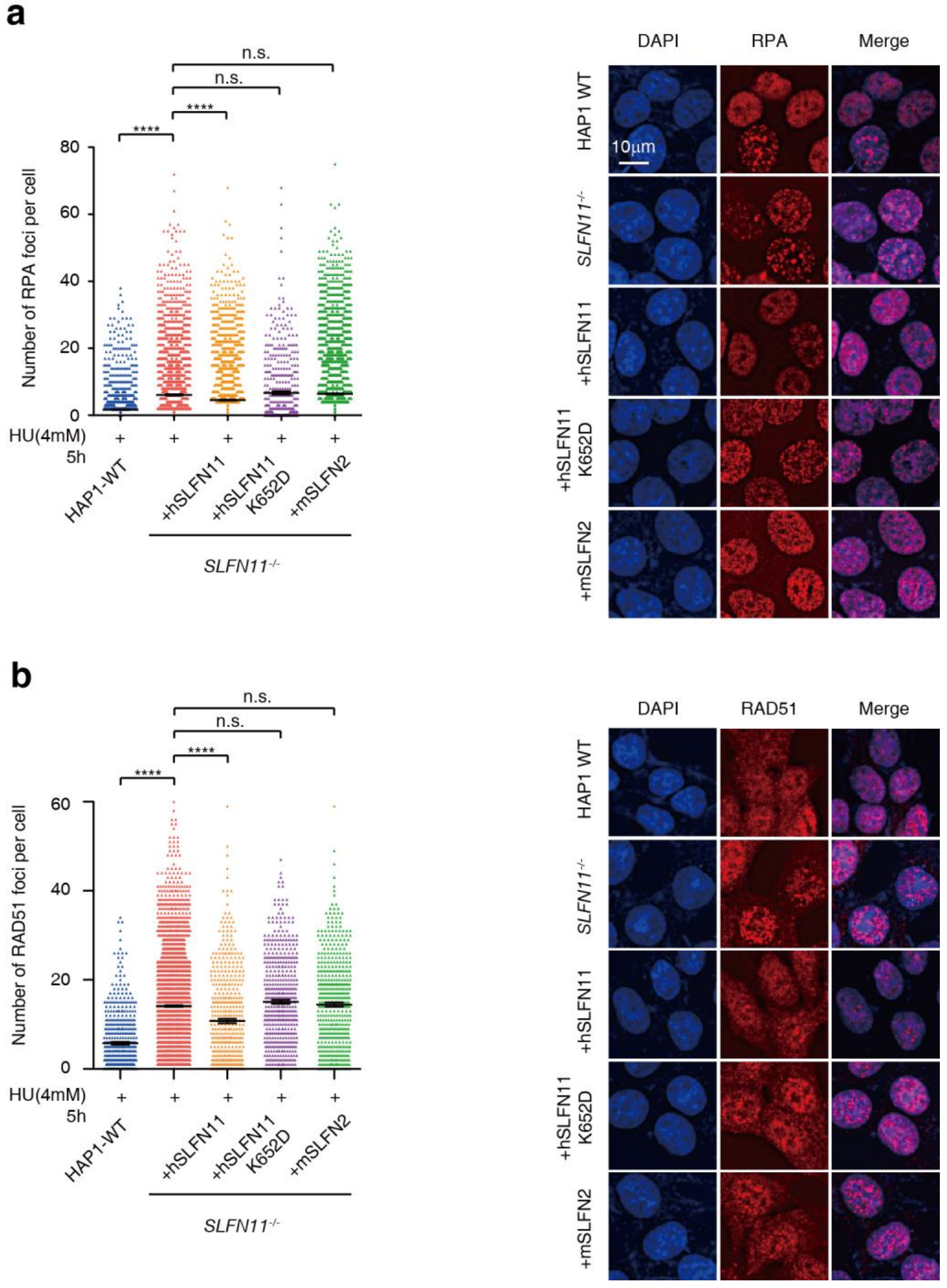

Supplementary Figure 8. RAD51 and RPA foci levels. Quantification of **a** RPA and **b** RAD51 foci per cell in HAP1 cell derivative with the indicated genotypes. Each dot represents the number of foci per nucleus in a single cell. Cells were exposed to HU 4mM for 5 hours and stained with the indicated antibodies. Mean ± SEM ($n \geq 500$) are shown for each condition. The experiment was repeated twice with similar results. The P values were calculated using one-way ANOVA with Tukey's multiple-comparisons test. Representative images are shown. n.s.: not significant. ****: *p<0.0001*

Figure 3a

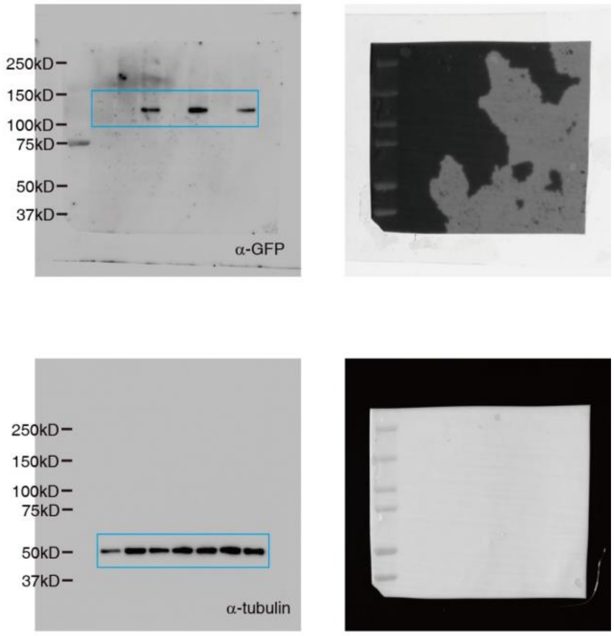

Figure 3f

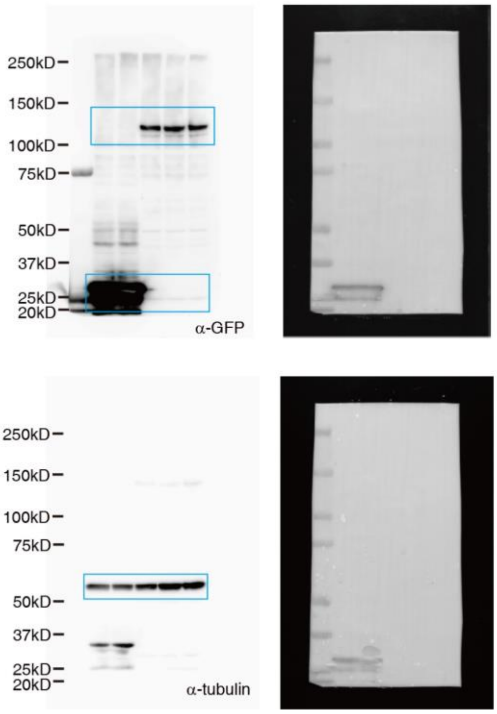

Supplementary Figure 9. **Uncropped Western blots.** Blots for Figure 3, as indicated.

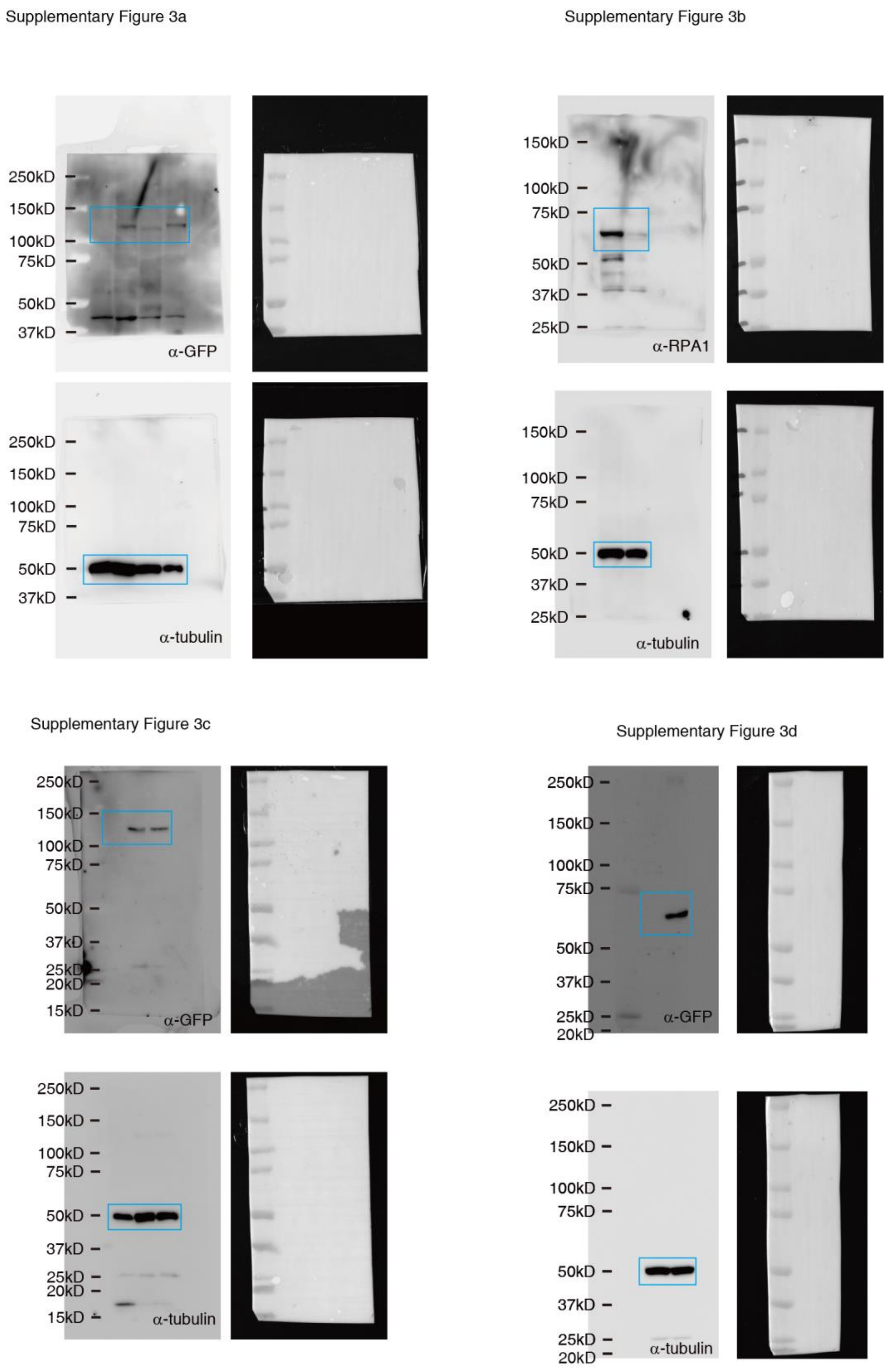

Supplementary Figure 10. **Uncropped Western blots.** Blots for Supplementary Figure 3, as indicated.

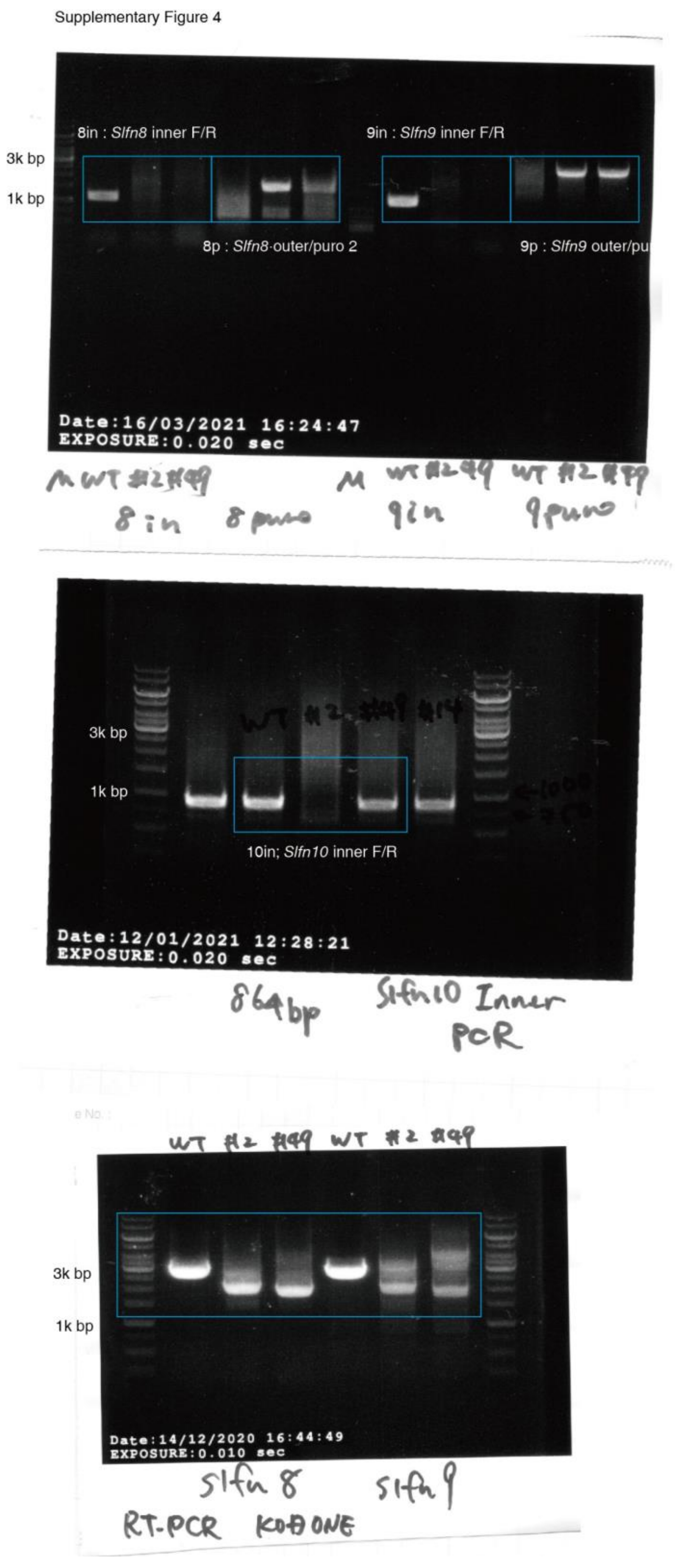

Supplementary Figure 11. **Uncropped gel images.** Gel images for Supplementary Figure 4, as indicated.

Supplementary Figure 5

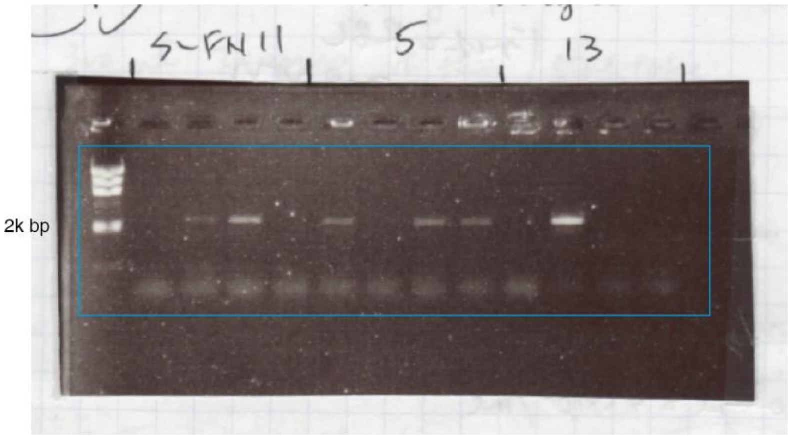

Supplementary Figure 12. **Uncropped gel image.** Gel image for Supplementary Figure 5, as indicated.

Supplementary Figure 6a

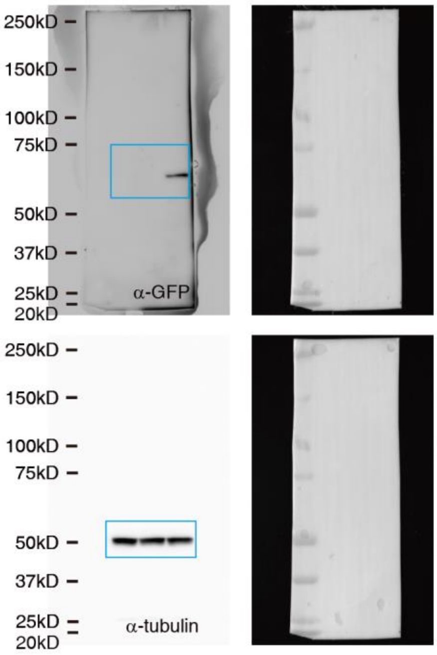

Supplementary Figure 6b

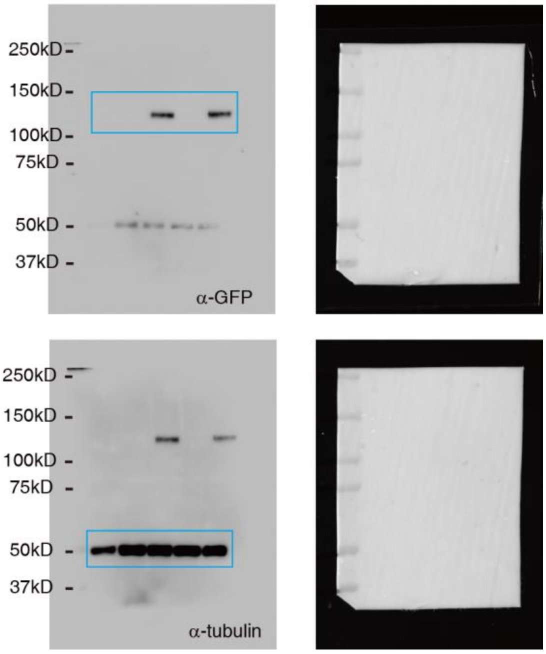

Supplementary Figure 13. **Uncropped Western blots.** Blots for Supplementary Figure 6, as indicated. There are remaining a-GFP or a-tubulin bands in these blots because of incomplete deprobing.

Supplementary Table 1. Antibodies used in this study

Name	Source	Catalog or REF No.	Dilution
anti-BrdU mouse mAb (B44)	BD Biosciences	347580	1:400
anti-BrdU rat mAb	Abcam	ab6326	1:400
anti-RPA2 mouse	Abcam	Ab2175	1:200
anti-RPA1 rabbit	Abcam	Ab79398	1:1000
anti-RAD51 rabbit polyAb	Dr. Hitoshi Kurumizaka		1:1000
anti-GFP mouse mAb	MBL	M048-3	1:1000
anti-mouse IgG F(ab')2-HRP	Amersham	NA9310V	1:2000
anti-rabbit IgG-Alexa Fluor 594	Invitrogen	A11037	1:500
anti-mouse IgG-Alexa Fluor 594	Invitrogen	A11032	1:500
anti-rat IgG-Alexa Fluor 488	Invitrogen	A11006	1:500

Supplementary Table 2. Primers used in this study

ID	Description	Sequence
KD20-42	*Slfn8* Forward	caccatggagacacatccctccttagcagt
KD20-43	*Slfn8* Reverse	gcatgcctccgttgagctatgcccctcagg
KD21-58	*Slfn9* Forward	gcaggctccgcggccaccatggagacatatctctccttagtg
KD23-1	*Slfn2* Forward	caccatgggtactagacttgaggcaactgagcaa
KD23-2	*Slfn2* Reverse	acctgatggggcattcatctggagtttcac
KD21-59	*Slfn9* Reverse	gcccaagcttgaattcgcatgccaccattgagctctgcccct
KD17-1	*SLFN11* Forward	gcaggctccgcggccgccaccatggaggcaaatcagtgccccctg
KD17-3	*SLFN11* Reverse	gcccaagcttgaattcatggccaccccacggaaaaatatac
KD20-10	*SLFN5* Forward	gcaggctccgcggccaccatgagtcttaggattgatgtg
KD20-11	*SLFN5* Reverse	gcccaagcttgaattccacagaagccttcagaatatacag
KD21-12	*SLFN13* Forward	gcaggctccgcggccaccatggaggcaaatcactgctcc
KD21-13	*SLFN13* Reverse	gcccaagcttgaattccagaaaaatatataggtgctgt
KD20-49	*Slfn9*arm Forward	gtcaagtatgtaatctcataggtg
KD20-50	*Slfn9*arm Reverse	taggtccctcgaagaggttcgagcgactagaggatttagt
KD20-48	*Slfn8*arm Reverse	tttttgacatcttcttccaagtata
KD20-49	*Slfn8*arm Forward	attaagggttattgaatatgacgtgtttggtagagaattg
KD22-57	*Slfn8* outer	tacgagccaaggaatctgggagct
KD22-58	*Slfn9* outer	ttttgttcaattccttcacctgtt

KD17-22	loxP *puro* 1	catattcaataacccttaat
KD17-23	loxP *puro* 2	gaacctcttcgagggaccta
KD20-60	*Slfn9* inner Reverse	aagttatgtggcagcagtgccacag
KD20-61	*Slfn9* inner Forward	caactcatatattgtttaaaatggagagtc
KD20-55	*Slfn8* inner Forward	gggacagccttgactgtcct
KD20-56	*Slfn8* inner Reverse	agtggcacatccaccacagg
KD20-70	*Slfn10* inner Forward	tctcccaaactgatgggcac
KD20-71	*Slfn10* inner Reverse	gggtaacgtggctcctgaaa

Supplementary References

1. Seita, J., Sahoo, D., Rossi, D. J., Bhattacharya, D., Serwold, T., Inlay, M. A., ... & Weissman, I. L. Gene Expression Commons: an open platform for absolute gene expression profiling. *PloS one*, **7**, e40321 (2012).
2. Yang, J. Y., Deng, X. Y., Li, Y. S., Ma, X. C., Feng, J. X., Yu, B., ... & Gao, S. Structure of Schlafen13 reveals a new class of tRNA/rRNA-targeting RNase engaged in translational control. *Nat. Commun.*, **9**, 1165 (2018).
3. Metzner,F.J., Huber,E., Hopfner,K.-P. and Lammens,K. Structural and biochemical characterization of human Schlafen 5. *Nucleic Acids Res.*, **50**, 1147-1161 (2022).
4. Metzner, F. J., Wenzl, S. J., Kugler, M., Krebs, S., Hopfner, K. P., & Lammens, K. Mechanistic understanding of human SLFN11. *Nat. Commun.*, **13**, 5464 (2022).
5. Mu,Y., Lou,J., Srivastava,M., Zhao,B., Feng,X. h, Liu,T., Chen,J. and Huang,J. SLFN11 inhibits checkpoint maintenance and homologous recombination repair. *EMBO Rep.*, **17**, 94-109 (2016).
6. Liu,F., Zhou,P., Wang,Q., Zhang,M. and Li,D. The Schlafen family: complex roles in different cell types and virus replication. *Cell Biol. Int.*, **42**, 2-8 (2017).
7. Bustos,O., Naik,S., Ayers,G., Casola,C., Perez-Lamigueiro,M.A., Chippindale,P.T., Pritham,E.J. and Casa-Esperón,E. de la Evolution of the Schlafen genes, a gene family associated with embryonic lethality, meiotic drive, immune processes and orthopoxvirus virulence. *Gene*, **447**, 1-11 (2009).
