# Supplementary material for: Mouse Slfn8 and Slfn9 genes complement human cells lacking SLFN11 during the replication stress response
Source: Commun Biol. 2023 Oct 13;6:1038. doi: 10.1038/s42003-023-05406-9 (PMC10575959; doi:10.1038/s42003-023-05406-9)
Supplement: Supplementary file 3 — Description of Additional Supplementary Files [file 42003_2023_5406_MOESM3_ESM.docx]

**Description of Additional Supplementary Files**

**File name:** Supplementary Data 1

**Description:** Numerical source data of graphs presented in the main figures.
