# Supplementary material for: Mouse Slfn8 and Slfn9 genes complement human cells lacking SLFN11 during the replication stress response
Source: Commun Biol. 2023 Oct 13;6:1038. doi: 10.1038/s42003-023-05406-9 (PMC10575959; doi:10.1038/s42003-023-05406-9)
Supplement: Supplementary file 5 — nr-reporting-summary [file 42003_2023_5406_MOESM5_ESM.pdf]

nature portfolio

Corresponding author(s): Anfeng Mu

Last updated by author(s): Sep 12, 2023

Reporting Summary

Nature Portfolio wishes to improve the reproducibility of the work that we publish. This form provides structure for consistency and transparency in reporting. For further information on Nature Portfolio policies, see our Editorial Policies and the Editorial Policy Checklist.

Statistics

For all statistical analyses, confirm that the following items are present in the figure legend, table legend, main text, or Methods section.

n/a	Confirmed	
☐	☒	The exact sample size (*n*) for each experimental group/condition, given as a discrete number and unit of measurement
☐	☒	A statement on whether measurements were taken from distinct samples or whether the same sample was measured repeatedly
☐	☒	The statistical test(s) used AND whether they are one- or two-sided<br>*Only common tests should be described solely by name; describe more complex techniques in the Methods section.*
☒	☐	A description of all covariates tested
☒	☐	A description of any assumptions or corrections, such as tests of normality and adjustment for multiple comparisons
☐	☒	A full description of the statistical parameters including central tendency (e.g. means) or other basic estimates (e.g. regression coefficient) AND variation (e.g. standard deviation) or associated estimates of uncertainty (e.g. confidence intervals)
☐	☒	For null hypothesis testing, the test statistic (e.g. *F*, *t*, *r*) with confidence intervals, effect sizes, degrees of freedom and *P* value noted<br>*Give P values as exact values whenever suitable.*
☒	☐	For Bayesian analysis, information on the choice of priors and Markov chain Monte Carlo settings
☒	☐	For hierarchical and complex designs, identification of the appropriate level for tests and full reporting of outcomes
☒	☐	Estimates of effect sizes (e.g. Cohen's *d*, Pearson's *r*), indicating how they were calculated

Our web collection on statistics for biologists contains articles on many of the points above.

Software and code

Policy information about availability of computer code

Data collection	Microplate Manager 6 Software, PerkinElmer 2030 Workstation, IN Cell Analyzer v4.6, Leica LAS AF software, Leica Application Suite X (LAS X) software
Data analysis	Graphpad prism v5.0f, Flow Cytometry Standard (FCS) 3.0, BLAT (https://genome.ucsc.edu/), IN Cell Developer Toolbox 1.9.2, 4Peaks v1.7.1, SnapGene Viewer

For manuscripts utilizing custom algorithms or software that are central to the research but not yet described in published literature, software must be made available to editors and reviewers. We strongly encourage code deposition in a community repository (e.g. GitHub). See the Nature Portfolio guidelines for submitting code & software for further information.

Data

Policy information about availability of data

All manuscripts must include a data availability statement. This statement should provide the following information, where applicable:

- Accession codes, unique identifiers, or web links for publicly available datasets
- A description of any restrictions on data availability
- For clinical datasets or third party data, please ensure that the statement adheres to our policy

All relevant data are available from the authors upon reasonable request.

Human research participants

Policy information about studies involving human research participants and Sex and Gender in Research.

Reporting on sex and gender	*Use the terms sex (biological attribute) and gender (shaped by social and cultural circumstances) carefully in order to avoid confusing both terms. Indicate if findings apply to only one sex or gender; describe whether sex and gender were considered in study design whether sex and/or gender was determined based on self-reporting or assigned and methods used. Provide in the source data disaggregated sex and gender data where this information has been collected, and consent has been obtained for sharing of individual-level data; provide overall numbers in this Reporting Summary. Please state if this information has not been collected. Report sex- and gender-based analyses where performed, justify reasons for lack of sex- and gender-based analysis.*
Population characteristics	*Describe the covariate-relevant population characteristics of the human research participants (e.g. age, genotypic information, past and current diagnosis and treatment categories). If you filled out the behavioural & social sciences study design questions and have nothing to add here, write "See above."*
Recruitment	*Describe how participants were recruited. Outline any potential self-selection bias or other biases that may be present and how these are likely to impact results.*
Ethics oversight	*Identify the organization(s) that approved the study protocol.*

Note that full information on the approval of the study protocol must also be provided in the manuscript.

Field-specific reporting

Please select the one below that is the best fit for your research. If you are not sure, read the appropriate sections before making your selection.

☒ Life sciences ☐ Behavioural & social sciences ☐ Ecological, evolutionary & environmental sciences

For a reference copy of the document with all sections, see nature.com/documents/nr-reporting-summary-flat.pdf

Life sciences study design

All studies must disclose on these points even when the disclosure is negative.

Sample size	No statistical methods were used to predetermine the sample size.
Data exclusions	No data was excluded.
Replication	All the experimental data were repeated more than twice and showed similar results.
Randomization	No randomization in this study.
Blinding	DNA fiber assay was carried out in a blinded manner.

Reporting for specific materials, systems and methods

We require information from authors about some types of materials, experimental systems and methods used in many studies. Here, indicate whether each material, system or method listed is relevant to your study. If you are not sure if a list item applies to your research, read the appropriate section before selecting a response.

Materials & experimental systems

n/a	Involved in the study	
☐	☒	Antibodies
☐	☒	Eukaryotic cell lines
☒	☐	Palaeontology and archaeology
☒	☐	Animals and other organisms
☒	☐	Clinical data
☒	☐	Dual use research of concern

Methods

n/a	Involved in the study	
☒	☐	ChIP-seq
☒	☐	Flow cytometry
☒	☐	MRI-based neuroimaging

Antibodies

Antibodies used	anti-BrdU mouse mAb (B44) BD Biosciences 347580<br>anti-BrdU rat mAb Abcam ab6326<br>anti-RPA2 mouse Abcam Ab2175<br>anti-RAD51 rabbit polyAb provided from Dr. Hitoshi Kurumizaka<br>anti-GFP mouse mAb MBL M048-3<br>anti-mouse IgG F(ab')2-HRP Amersham NA9310V<br>anti-rabbit IgG-Alexa Fluor 594 Invitrogen A11037<br>anti-mouse IgG-Alexa Fluor 594 Invitrogen A11032<br>anti-rat IgG-Alexa Fluor 488 Invitrogen A11006
Validation	anti-BrdU mouse mAb (B44) BD Biosciences 347580,<br>anti-BrdU rat mAb Abcam ab6326,<br>anti-RPA2 mouse Abcam Ab2175,<br>anti-RAD51 rabbit polyAb provided from Dr. Hitoshi Kurumizaka,<br>anti-GFP mouse mAb MBL M048-3, were used in many recent researches as described in the datasheets.

Eukaryotic cell lines

Policy information about cell lines and Sex and Gender in Research

Cell line source(s)	HAP-1 cell was purchased from Horizon Genomics.<br>hTERT RPE-1 was purchased from ATCC.<br>293T, HL60, and Ba/F3 cells were from the stock of Takata lab.
Authentication	HAP-1 and hTert1-RPE1 cells were obtained from authenticated source.
Mycoplasma contamination	The cell lines were tested for mycoplasma contamination before we received them from the cell bank. We are using RT-PCR for mycoplasma contamination testing with HAP-1 cells.
Commonly misidentified lines<br>(See ICLAC register)	No commonly misidentified cell lines were used in this study.
